# Blebs and former blebs: From surface protrusions to extracellular vesicles in cancer signalling, anoikis resistance and beyond

**DOI:** 10.1002/jex2.112

**Published:** 2023-10-19

**Authors:** Dolores Di Vizio, Melanie Schoppet, Ashani Weeraratna, Kenneth W. Witwer

**Affiliations:** ^1^ Department of Surgery, Division of Cancer Biology and Therapeutics Cedars‐Sinai Medical Center Los Angeles California USA; ^2^ Independent Researcher; ^3^ Department of Biochemistry and Molecular Biology Johns Hopkins Bloomberg School of Public Health Baltimore Maryland USA; ^4^ Department of Oncology, Sidney Kimmel Cancer Center Johns Hopkins University School of Medicine Baltimore Maryland USA; ^5^ Department of Molecular and Comparative Pathobiology Johns Hopkins University School of Medicine Baltimore Maryland USA; ^6^ Department of Neurology Johns Hopkins University School of Medicine Baltimore Maryland USA; ^7^ Richman Family Precision Medicine Center of Excellence in Alzheimer's Disease Johns Hopkins University School of Medicine Baltimore Maryland USA

**Keywords:** anoikis, cancer, ectosomes, exosomes, extracellular matrix, extracellular vesicles, large oncosomes, melanoma, membrane blebbing, metastasis, septin, tumour microenvironment

## Abstract

Associations between plasma membrane blebbing and metastatic progression have been widely reported. There are also reports of increased extracellular vesicle (EV) release from cancer cells. Yet the ties between these closely related phenomena are incompletely understood. In this commentary, we remark on a recent finding on cellular membrane blebs in melanoma signalling. We discuss possible implications for cancer biology and draw parallels to knowns and unknowns in the relationships of EVs and cancer progression.

## 1 MEMBRANE BLEBS, EXTRACELLULAR VESICLES AND CANCER SIGNALLING

Weems, Danuser and colleagues recently proposed cellular membrane blebs as ‘oncogenic signalling hubs’ (Weems et al., 2023), raising important questions about cellular and extracellular membranes in cancer and beyond. Membrane blebbing was first reported in 1923 by Margaret Reed Lewis, studying chick embryo cultures (Lewis, 1923). In blebbing, the cell membrane undergoes local deformation and outward bubble‐like protrusion, influenced by membrane tension and the local state of the actin cytoskeleton (Charras, 2008). In turn, these factors take external and internal cues from mechanical stress, chemical gradients and more. Blebs can be quite large in diameter, in the micron range, but they can also be much smaller. Healthy cells form blebs to assist with migration and support cell division (Fackler & Grosse, 2008; Paluch & Raz, 2013; Paluch et al., 2006). Increased levels of membrane blebbing are strongly associated with tumour invasion and metastasis, as well as with apoptosis (Sekyrova et al., 2012; Vermeulen et al., 2005).

The connection of membrane blebs to cancer signalling starts with the observation that most mammalian cells require attachment to the extracellular matrix for survival. Without this anchorage and specifically the constant signalling reinforcement it provides, cells tend to undergo a cell death process known as ‘anoikis’ (reviewed in (Frisch & Screaton, 2001; Simpson et al., 2008; Taddei et al., 2012)). For a cancer cell to leave its substrate and live to tell the tale (i.e., metastasize), it must overcome the anoikis problem. In melanoma, the BRAF oncogene (serine/threonine‐protein kinase B‐Raf) is often mutated, and signalling through this pathway has been shown to confer resistance to anoikis. Since BRAF is a driver oncogene in melanoma, this suggests that anoikis resistance is a key feature of melanoma metastasis (Boisvert‐Adamo & Aplin, 2008).

To find out whether membrane blebs—well known to decorate metastatic cells—also contribute to anoikis resistance, Weems et al. treated melanoma cells to inhibit blebbing (Weems et al., 2023). One approach was to treat cells with wheat germ agglutinin (WGA), a lectin that binds to the glycocalyx and thereby minimizes membrane deformation. The second approach used a hydrogel to stop blebs from growing outward. The results were clear: impeding bleb formation kept cells from developing anoikis resistance. Next, the authors found that the characteristic membrane curvature of blebs was responsible for local enrichment of septins, a class of proteins that, among other roles, sense membrane curvature and promote membrane constriction by regulating actomyosin‐based contractility (Founounou et al., 2013; Mostowy & Cossart, 2012; Szuba et al., 2021), for example, in cytokinesis. Septins were determined by Weems and colleagues to be necessary for development of anoikis resistance, a finding that was underlined not only through treatment with general blebbing impeders, but also with Rho‐associated protein kinase (ROCK) and Ezrin inhibitors. The septin inhibitor forchlorfenuron also abrogated anoikis resistance: importantly, without preventing actual bleb formation. Finally, septins were found to scaffold NRAS (N‐ras proto‐oncogene), which could then achieve pro‐survival signalling via pathways involving phosphoinositide 3‐kinases and AKR thymoma/protein kinase B (PI3K‐AKT) and RAF–MEK–ERK (rapidly accelerated fibrosarcoma—MAPK/ERK—extracellular signal‐regulated kinases). Blebs—or more precisely their recruited septins and their binding partners—thus appear to be responsible for anoikis resistance.

The remarkable findings of Weems et al. have major implications for cancer studies and therapeutics; we posit that they also raise numerous interesting questions about extracellular vesicles (EVs). As lipid bilayer‐delimited particles that are released by all known cells, EVs have been classically categorized by biogenesis into plasma membrane‐origin ‘ectosomes’ (aka microvesicles, microparticles) and endosomal‐origin ‘exosomes’ (Buzas, 2023; Cocucci & Meldolesi, 2015; György et al., 2011). Although presumed exosomes have garnered overwhelmingly more research attention than other EVs, this may be due to historical biases and misconceptions (Witwer & Théry, 2019). We now recognize, for example, that ectosomes can be just as small as exosomes; that there is no simple way to separate members of the two classes after they have left the cell; that ectosomes may actually outnumber exosomes; and that ectosomes may have functions that are at least as consequential as those of exosomes (Buzas, 2023; Magoling et al., 2023; Mathieu et al., 2021; Minciacchi et al., 2017; Théry et al., 2018). Importantly, every ectosome is a former plasma membrane bleb.

We recall an iconic electron micrograph of a gnarly‐looking glioblastoma cell with protrusions extending in all directions, published by Johan Skog and Xandra Breakefield's group in 2008 (Skog et al., 2008) and since shown in uncounted presentations on EVs and cancer. In the figure, portions of the protrusions and cell body alike are covered with apparent EVs of around 50–100 nm in diameter. Skog et al. reported that cancer cell EVs spread both RNAs and proteins from the cell of origin, implicating EVs and their molecular cargo as biomarkers of cancer. Furthermore, cancer EVs could promote cell proliferation and angiogenesis, both important for tumour growth. Ectosomes were also identified by Janusz Rak's team as shuttles of the EGFR variant EGFRvIII between glioma cells (Al‐Nedawi et al., 2008); by Crislyn D'Souza‐Schorey's group as proteolytic platforms in cancer that are regulated by ARF6 (Muralidharan‐Chari et al., 2009); and by Michael Dustin and collaborators as important effectors at the immunological synapse (Saliba et al., 2019). These papers and others expanded the growing realization of the functional roles of EVs and remind us today that many EVs form—and may remain—at the cell surface.

Small EVs like those depicted in the Skog et al. glioblastoma micrograph are by no means the only PM‐origin EVs. ‘Large oncosomes’ of up to several microns in diameter—and thus consistent in size with the blebs observed by Weems et al.—were first described by Di Vizio and colleagues in 2009 (Di Vizio et al., 2009). Highly metastatic prostate cancer cells formed and released non‐apoptotic blebs after losing the actin nucleator and cytoskeleton regulator Diaphanous‐related formin 3 (DIAPH3). With the deletion of DIAPH3, cell motility switched to a Rho‐GTPase/ROCK‐mediated amoeboid mode of rapid, propulsive motion requiring limited or no proteolysis (Hager et al., 2012). While the mechanisms that control the amoeboid phenotype in tumour cells remain poorly understood, as does the connection with anoikis resistance in malignant progression, follow‐up studies have firmly established the association of large oncosomes and tumour progression (Di Vizio et al., 2012; Gerdtsson et al., 2021; Meehan et al., 2016; Minciacchi et al., 2017; Zhang et al., 2022).

## 2 QUESTIONS AND DIRECTIONS

We submit that a host of important questions about EVs and cancer signalling are raised by the findings of Weems and colleagues, some of which we list below. Of note, we are currently unable to give satisfying answers to many of these questions, but we hope that they are provocative and will stimulate further thought and investigation.

### 2.1 What is the relationship between bleb formation and EVs, and how might it change during oncogenesis?

How frequently is a membrane bleb released as an ectosome? Do all membrane blebs have the same potential to be released? Or are some bleb formation mechanisms more or less likely to allow release? Are the processes different for different cancer types? If different sizes of blebs/EVs, (e.g., the large blebs reported by Weems et al. and the small EVs shown by Skog et al.) are observed on the surfaces of different types of cells or stages of metastatic progression, what might underly these differences? When are energy‐consuming processes involved in bleb scission/release, and can we inhibit or promote them? Additionally, given the importance of BRAF signalling in melanoma pathogenesis, can blebs contribute to therapy resistance? Is a cell with more blebs inherently more resistant to BRAF‐targeted therapies, and what can be done to overcome this resistance?

### 2.2 How ‘portable’ are septins and their signalling effects?

**FIGURE 1:**
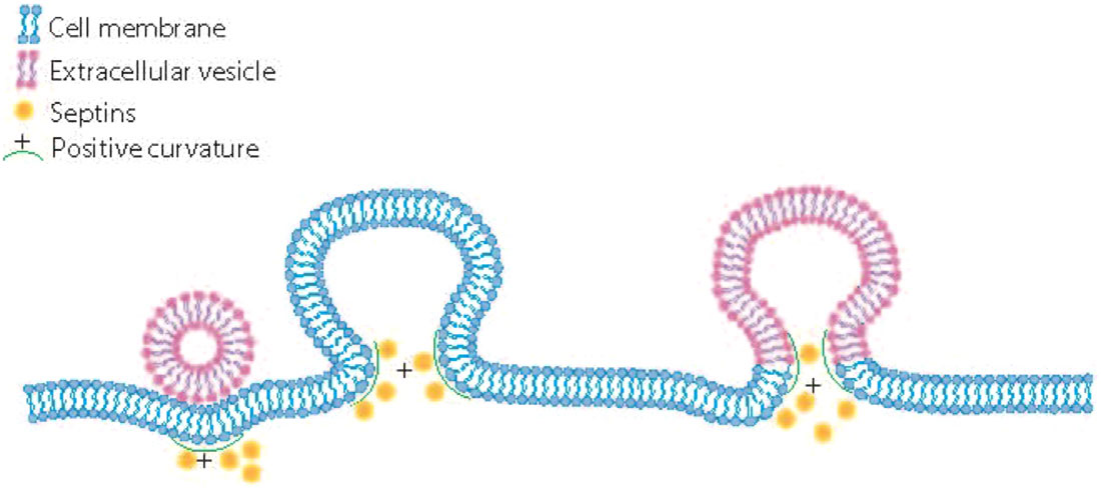
Blebs, extracellular vesicles (EVs) and membrane curvature. Weems, et al., report that blebbing induces positive membrane curvature and assembly of septins as a signalling platform (blue membrane bleb). Potentially, cells might also experience positive local membrane curvature when they encounter extracellular particles (left) or when, for example, large EVs fuse with a cellular membrane (right).

Could blebs that are released from one cell recapitulate anti‐anoikis effects in a recipient cell? Xu and colleagues found septins to be enriched in larger versus smaller EVs (Xu et al., 2015); could septins or their binding partners be carried from one cell to another by EVs? Is septin‐mediated signalling promoted only during blebbing, or also by EV association with the plasma membrane? In the Weems et al. findings, septins are enriched in domains that appear to be stabilized by positive curvature, that is, the inward curvature of the plasma membrane that occurs between blebs (Weems et al., 2023). Might septin enrichment also occur during EV‐cell fusion, or even without fusion, if an EV presses against and locally deforms the PM (Figure 1)? This would be yet another way for an EV to affect a cell without membrane‐membrane fusion. Finally, since septins associate with the membrane and are known to be involved in, for example, cytokinesis, are septins involved in the scission of membrane blebs from the membrane, thus forming EVs?

### 2.3 Below the surface: What is happening with septins further inside the cell?

At least in some settings, cancer cells reportedly release not only more ectosomes, but also more exosomes from the multivesicular body (MVB). From the perspective of the cytoplasm, the MVB membrane and an intermediate fusion product of the MVB and PM have positive curvature. In this light, does increased exosome release also lead to septin domain formation and stabilization?

### 2.4 When is a bleb not a bleb?

It is not always possible to distinguish a cell‐surface bleb from a surface‐associated EV, and the latter could have one of several origins. It might be a former PM bleb that has only transiently ‘released’ from the parent cell surface or has associated with a second cell after release from another. It could also be an exosome from the same or a different cell that adheres after fusion of the MVB with the cell surface. Finally, molecularly and mechanistically, is there anything special about a bleb that releases as an ectosome but then back‐fuses?

### 2.5 Apart from potential roles in anoikis resistance, what business do EVs have on the surface of cells?

EV depletion from cell culture media stimulates lipid synthesis pathways in cells (Liao et al., 2017), and this may be one possible factor contributing to the widely reported phenomenon of EV release upon cell starvation. Do EVs from around the cell associate with the cell surface and act as a kind of ‘security blanket’ for the cell? For example, by buffering access to signalling pathways? By contributing an outer perimeter of antimicrobial defence? For other reasons?

## 3 CONCLUSION

One hundred years after the first descriptions of membrane blebs (Lewis, 1923), our understanding of their importance in biological systems continues to evolve in fascinating ways. Some blebs retain continuity with the cell membrane. It was these blebs that revealed to Weems et al. their vital role in anoikis resistance of melanoma cells (Weems et al., 2023). But other blebs mature to dissociate from the parent cell membrane, thus becoming ectosomes: ranging from small EVs to large oncosomes. Whether released from the surface as ectosomes or secreted as exosomes, EVs may continue to associate with the parent cell or drift away to interact with distant cells, either with new surface interactions or by membrane‐membrane fusion. The time is ripe to learn more about EVs as erstwhile blebs and the functional consequences they may present for both parent and recipient cells.

## AUTHOR CONTRIBUTIONS

**Dolores Di Vizio**: Conceptualization; writing—review and editing. **Melanie Schoppet**: Conceptualization; visualization; writing—review and editing. **Ashani Weeraratna**: Conceptualization; writing—review and editing.

## CONFLICT OF INTEREST STATEMENT

M.S. has shares of Exopharm, Ltd. K.W.W. has or has had sponsored research agreements with Ionis Pharmaceuticals, Yuvan Research and AgriSciX; is or has been an advisory board member of ShiftBio, Exopharm, NeuroDex, NovaDip and ReNeuron; holds stock options with NeuroDex; and performs ad hoc consulting as Kenneth Witwer Consulting.
